# 

**Published:** 1894-11-03

**Authors:** 


**Notices of Births, Deaths, and Marriages** are inserted in this column at a charge of 2s. 6d. for an announcement not exceeding four lines.

**Notice to Advertisers and Subscribers.**

All Advertisements, Orders for Copies of THE HOSPITAL, and Business Communications should be addressed to **The Publisher, NOT TO THE EDITOR,** THE HOSPITAL, 428, Strand, W.C.

The Cost of Subscription, post free, is as follows:—Per week, 2½d.; per quarter, 2s. 8d.; per half-year, 5s. 3d.; per annum, 10s. 6d. Foreign:—Per Annum, 12s. 8d.; payable, in all cases, in advance.

**NOTICE TO CORRESPONDENTS.**

All MS., Letters, Books for Review, and other matters intended for the Editor should be addressed THE EDITOR, THE LODGE, PORCHESTER SQUARE, LONDON, W.

The Editor cannot undertake to return rejected MS., even when accompanied by stamped directed envelope.

**"Burdett's Hospital Annual," 1894.**

Fifth year of publication. 900 pp. Boards, gilt lettering. A most complete guide to British, Colonial, Indian, and American Hospitals, Dispensaries, Nursing and Convalescent Institutions, and Asylums. Price 5s. Published by THE SCIENTIFIC PRESS (Limited), 428, Strand, W.C.

**NOTICE.—The Index for Vol. XVI. (ending September 24th, 1894) is on sale at the Office of this Journal, Price 2½d., post free.**

### SCRAPS AND GLEANINGS.

Last week the German Hospital at Dalston celebrated its jubilee.

The Archbishop of York has accepted the presidency of the Church Sanitary Association.

Mr. Gladstone has given £20 towards the building fund of the Royal London Ophthalmic Hospital.

Sir Joseph Lister is to be presented with a memorial on the occasion of his retirement from active work.

The Porth Semi-National Eisteddfod Committee has contributed £423 towards the funds being raised for the Porth Cottage Hospital.

A bequest is announced as having been received at the Jervis Street Hospital, Dublin, from the late Mrs. Elizabeth Ryan. The sum amounts to £300.

During the year under review the Croydon General Hospital has received 11,000 persons as patients. This institution is doing a good useful work.

The French Academy of Moral and Political Sciences has bestowed the Audiffret prize of £480 on Dr. Roux, for his discovery of a remedy for diphtheria.

The committee of the Darlington Hospital are seriously considering the advisability of increasing the adult accommodation and adding a children's wing.

The Apollinaris Company (Limited) announce that on and after November 1st, 1894, their address will be 4, Stratford Place, Oxford Street, London, W.

The extension which was recently effected at the Royal Free Hospital, Gray's Inn Road, cost £28,500. Towards this sum £22,500 has already been received in contributions.

The Whitby Local Board have proposed to borrow £2,500 for the purchase of Fairfield Farm, Hawsker, as the site for a hospital for infectious diseases and other purposes.

The position of dental surgeon to the Institute for Deaf and Dumb at New York has been filled for some time past by a woman. Dr. Caroline Benton has the charge here of 350 patients.

The Archbishop of Canterbury presided at the opening of the new wing of the Croydon General Infirmary. His Grace commented on the interest shown of late by the working men in the success of their hospital.

The report of the past year's work at St. Mary's Hospital, Manchester, has been satisfactory. The number of patients has largely increased, some 17,000 persons having been treated. The income has been steadily maintained.

The formal opening of the Retford Dispensary and Cottage Hospital has just taken place, the present establishment taking the place of the Jubilee one, now too small to fulfil the ever-increasing demands of the neighbourhood.

A donation of £50 has been received by the authorities of the Norfolk and Norwich Hospital from Major G. N. Micklethwaith, of Taverton, and £20 from the Worshipful Company of Mercers, out of the funds of the Earl of Northampton's Charity.

The annual report of the Plymouth Public Dispensary states that during the twelve months under review 3,721 patients have been treated. The finances are in a fairly satisfactory condition, the adverse balance being about £60 at the close of the year.

The North-West London Hospital, Kentish Town Road, has received, through the treasurer, Mr. Herring, £100 from the "Children's Salon," in connection with the *Gentlewoman*, towards the endowment of a cot. Mr. Herring has added £100 to this amount.

A Bronze statue has been erected and recently unveiled in New York in commemoration of Dr. J. Marion Sims. This movement, which was set on foot by the "New York Medical Record," has enlisted subscriptions for the necessary funds from members of the medical profession of America and other countries.

It has been now definitely announced by the chairman of the St. Mary's Hospital, Manchester, that its proposed amalgamation with the Southern Hospital has been practically settled, subject to the approval of the Charity Commissioners, and £70,000 has been set aside of the David Lewis trust for this purpose.

The Malvern Local Board has obtained a site of two and a-half acres for the proposed joint isolation hospital for the district, and a contract has been entered into with Messrs. Humphreys, of London, to put up an iron hospital lined with felt and match-boarding at a cost of £280, and the buildings will be ready for use in a month's time.

Colonel Seeley, M.P., has made a munificent gift to the Nottingham General Hospital, the 112th anniversary of which has just been celebrated. Following on his donation of £1,000 in 1891 for convalescent work, he has now bought a mansion and ground to be converted into a convalescent home. The capitalized value of the gift is estimated at £20,000.

Sunday last was observed as Hospital Sunday in Brighton, and fortunately the day was brilliantly fine. Special sermons were preached in almost every church and chapel. The amount at present collected has reached the sum of £1,002 14s., as against £1,368 18s. last year. A few churches and chapels have yet to make their special collection.

A report by the College of Physicians of Edinburgh on the various possible sites for the New City Hospital has been circulated among the members of the Public Health Committee of the Edinburgh Town Council. Much difficulty is still being experienced as to the final decision of this question of the site.

With the object of avoiding the possibility of disease contagion through the medium of the public telephone mouthpiece, a German inventor has originated a very simple preventative This is the construction of a pad with a large number of discs of paper, with a hole in the centre, the upper disc of paper being torn off as each person uses it.

It is proposed to add a new wing to the Westminster Hospital for the treatment of female cancer patients, towards which funds are earnestly entreated. A sum of £4,000 is still required to carry out the same. The treatment of cancer has always been a special feature of this hospital, and the accommodation has now proved insufficient to meet all demands.

### BOOKS RECEIVED.

T. DOBB and Co., LIVERPOOL.

"Notes on Nursing." By Nurse Evelyn Herbert.

THE RELIGIOUS TRACT SOCIETY.

"The Sanitary Code of the Pentateuch." By the Rev. C. G. K. Gillespie.

MACMILLAN AND CO.
"The Preservation of Health in India." By Sir J. Fayrer, K.C.S.I., &c.

BALLIERE, TINDALL, AND COX.

"Indian Medico-Chirurgical Review."

SIMPKIN, MARSHALL, AND CO.
"Wright's Physicians, Surgeons, and Consultants' Visiting List, 1895.

H. K. LEWIS.
"Ambulance Lectures: First Aid to the Injured." By S. Osborne, F.R.C.S.

THE SCIENTIFIC PRESS.
"Spinal Curvature and Awkward Deformity: Their Causes and Prevention in Children." By Dr. George Müller. Edited and adapted by Richard Greene, F.R.C.P.

SOCIETY FOR PROMOTING CHRISTIAN KNOWLEDGE.
"Edible and Poisonous Mushrooms." By M. C. Cooke, M.A., LL.D.
"Our Secret Friends and Foes." By Percy Faraday Frankland, F.R.S. New Edition.
"Fruit Culture for Profit." By C. B. Whitehead, B.A.

WHITTAKER AND CO.

"Dissections Illustrated." By C. Gordon Brodie, F.R.C.S. Part III.—The Head, Neck, and Thorax.

YOUNG J. PENTLAND.
"First Aid to the Injured." By E. J. Lawless, M.D.

**Periodicals and Pamphlets.**—Journal d'Hygiène, The Rural World, Journal de Medicine, New York Medical Record, Public Health, The Strand Magazine, The Picture Magazine, The Million, Tits-Bits, Revue Genérale des Sciences, The Scientific World, Health Record, The Literary Digest, Guy's Hospital Gazette, The Corpuscle, The Monthly Homœopathic Review, Charity Organization Review, Indian Engineering, The Planter.

### THE STUDENT OF MEDICINE.

### APPOINTMENTS.

L. W. Bathurst, M.B.Lond., M.R.C.S., L.R.C.P., appointed Assistant Medical Officer at the London County Asylum, Hanwell. H. S. Collier, F.R.C.S., appointed a Demonstrator of Anatomy at St. Mary's Hospital Medical School. W. R. Dawson, M.D., &c., Dub., appointed Assistant Medical Superintendent to Farnham House and Mary Ville Private Asylums, Finglas, Dublin. P. S. Eves, M.B., B.S., appointed House Surgeon to the Cumberland Infirmary, Carlisle. W. E. Fothergill, M.A., B.Sc., M.B., C.M., appointed House Surgeon to the Edinburgh Royal Maternity and Simpson Memorial Hospital. L. J. Godson, M.R.C.S., L.R.C.P., appointed House Surgeon to the Royal United Hospital, Bath. E. P. Isaacs, M.R.C.S., L.R.C.P., appointed House Surgeon to the Dreadnought Hospital, Greenwich. W. D. Lawrie, M.B., C.M., appointed Junior House Surgeon to the Branch of the Seamen's Hospital, Royal Albert Dock, E. E. W. Martland, M.R.C.S., L.R.C.P.Edin., L.S.A., appointed Honorary Surgeon to the Oldham Infirmary; W. Nicholson, M.B., C.M.Glas., appointed Superintendent and Resident Medical Officer to the Victoria Infirmary of Glasgow. R. Nitch Smith, M.R.C.S.Eng., L.R.C.P.Lond., appointed Medical Officer to the *Warwick Castle* under Government transport orders. J. M. Rutherford, M.B., C.M.Edin., appointed Assistant House Surgeon to the Cumberland Infirmary, Carlisle. G. A. G. Simpson, M.R.C.S., L.S.A., appointed Medical Officer of Health to the Acton Local Board. W. A. Stephen, M.A., M.B., C.M., appointed House Surgeon to the Edinburgh Royal Maternity and Simpson Memorial Hospital. W. A. Stott, M.R.C.S., L.R.C.P., late Resident Obstetric Officer, appointed Pathological Curator to the Leeds General Infirmary. G. W. Till, L.R.C.P.Edin., L.F.P.S.Glas., appointed Medical Officer of the Cottage Homes of the Worcester Union. A. H. Whicher, M.R.C.S.Eng., appointed Medical Officer for the Clutton District of the Clutton Union. C. W. Windsor, M.R.C.S., M.A., M.B., B.S., appointed House Physician to the Dreadnought Hospital, Greenwich.

### VACANCIES.

#### Resident Medical Officers.

East London Hospital for Children, Glamis Road, Shadwell, E.—House Surgeon. No salary. Board, lodging, and washing provided. Applications and testimonials to Thomas Hayes, Secretary, before November 10th.

Hospital for Women and Lying-in Institute, Brighton.—House Surgeon; unmarried, and under 30 years of age. Salary £80 per annum, with board and rooms. Applications to the Secretary by November 15th.

Ratcliffe Infirmary, Oxford.— House-Physician and House-Surgeon. Salary, £80 per annum, with board, lodging, and washing. Tenable for six months. Applications and testimonials to A. C. Virgo, Secretary, by November 16th.

THE STUDENT OF MEDICINE—*continued.*

**Non-Resident Medical Officers.**

Central London Ophthalmic Hospital, Gray's Inn Road, W.C.—Two Assistant Surgeons. Applications to the Secretary by November 5th.

County of Lanark.—Medical Officer; applicants must be under 50 years of age and registered medical practitioners; must hold diploma in Sanitary Science, Public Health, or State Medicine. Salary, £700 per annum, with travelling expenses and allowance for clerk and office. Applications and testimonials to W. Alston Dykes, County Clerk, before November 5th.

Glasgow Maternity Hospital.—Indoor and Outdoor Surgeons. Applications to Arthur Forbes, Secretary, 146, Buchanan Street, Glasgow, by November 7th.

Sunderland Infirmary.—Honorary Physician. Applications to the Chairman of the Medical Board by November 5th.

Hulme Dispensary, Manchester.—Honorary Surgeon. Applications to C. C. Heywood, Esq., F.R.C.S., Honorary Secretary, Medical Committee, by November 5th.

North-Eastern Hospital for Children, Hackney Road, Shoreditch, N.E. —Physician; must be F. or M.R.C.P.Lond. Applications to T. Glenton-Kerr, Secretary, 27, Clement's Lane, E.C., by November 15th.

North London Hospital for Consumption, Hampstead.—Assistant Physician. Candidates must be graduated in Medicine of a University of the United Kingdom or Fellows or Members of the Royal College of Physicians of London. Applications to L. F. Hill, M.A., Secretary, at the Office, before November 3rd.

Royal Westminster Ophthalmic Hospital, King William Street, West Strand, W.C.—Two Assistant Surgeons. Applications to T. Beattie-Campbell, Secretary, by November 3rd.

